# Allergic diseases of the skin and drug allergies – 2004: Clinical and immunologic characteristics of allergen-specific immunotherapy in children with atopic dermatitis

**DOI:** 10.1186/1939-4551-6-S1-P94

**Published:** 2013-04-23

**Authors:** Tatiana Slavyanskaya, Vladislava Derkach

**Affiliations:** 1 Medical Faculty, Postgraduate Course, University of Russia, Moscow, Russia; 2 Vladivostok State Medical University, Vladivostok, Russia

## Background

The aim was to determine the dynamic of clinical symptoms and saliva concentrations (SC) of IL-4, IL-13, IFNγ in children (Ch) with atopic dermatitis (AD) in the setting of background therapy (BT) and accelerated parenteral allergen-specific immunotherapy (APAI) with house dust mite allergens (HDMA).

## Methods

The study included a total of 33 Ch with non-acute AD. The mean age at enrollment was 7.2±2.1. The APAI has been provided for 36 months (M) according to accelerated regimen by parenteral introduction of HDMA (2 times/daythrough 6 h.), along with BT. SC of IL-4, IL-13, IFNγ were measured using ELISA at 1, 3, 6, 12 and 36M after treatment initiation.

## Results

The efficacy after 12M of APAI was 78.7–84.8% depending on disease severity. The “excellent” result after APAI course was achieved in 21.2% Ch with AD, “good” in 57.6% Ch, “satisfactory” in 9.1% Ch, and there was no therapeutic effect in 12.1% Ch. There was reduction in the number, duration and severity of AD exacerbations observed in 2/3 Ch, which allowed the subsequent reduction of BT. As early as at 6 M there was a 1.5 times decrease of hospitalization number (p<0.05) as compared to the pre-treatment year. After the 1st M of APAI in Ch with AD there was no significant decrease of SC of IL-4, IL-13 and no increase of saliva IFNγ level either (p<0.05). There was no normalization of SC IL-4 and IL-13 during first 6 months in Ch with severe AD as well, though their levels decreased at an average of more than 1.5 times. 12 M of APAI provided duplication of local SC of IFNγ. This saliva IFNγ increase in Ch with AD on the background of APAI was accompanied by decrease of specific IgE againstHDMA (r= –0.73). The pre-APAI immune disorder formula was IL-43+IL-132+IFNγ3-IgE3+. After termination of treatment course withHDMA it has changed for IL-42+IL-132+IFNγ1-IgE2+.

## Conclusions

The results showed that pathogenetic therapy contributes to reduction of SC and inhibits immune inflammation by down regulating the pro-inflammatory cytokine IL-4 and IL-13 production and activating IFNγ synthesis. Thus the accelerated parenteral APAI is effective in Ch with AD.

